# Composition and Element Distribution Mapping of γ′ and γ″ Phases of Inconel 718 by High-Resolution Scanning Transmission Electron Microscopy and X-ray Energy-Dispersive Spectrometry

**DOI:** 10.3390/ma17030594

**Published:** 2024-01-26

**Authors:** Philippe A. Buffat, Ioannis Alexandrou, Aleksandra Czyrska-Filemonowicz

**Affiliations:** 1 Ecole Polytechnique Fédérale de Lausanne, Centre Interdisciplinaire de Microscopie Electronique, Ch. des Vioz 14, 1865 Les Diablerets, Switzerland; 2 Thermo Fisher Scientific, De Schakel 2, 5651 GH Eindhoven, The Netherlands; ioannis.alexandrou@thermofisher.com; 3 Faculty of Metals Engineering and Computer Science, Centre of Electron Microscopy for Materials Science, AGH University of Science and Technology, al. A. Mickiewicza 30, 30-059 Krakow, Poland; czyrska@agh.edu.pl

**Keywords:** superalloys, Inconel, interface structure, phase composition, EDXS-HRSTEM

## Abstract

The main strengthening mechanism for Inconel 718 (IN718), a Ni-based superalloy, is precipitation hardening by γ′ and γ″ particles. It is thus essential, for good alloy performance, that precipitates with the desired chemical composition have adequate size and dispersion. The distribution of the γ′ and γ″ phases and their chemical composition were investigated in the nickel-based Inconel 718 superalloy by taking advantage of the new capabilities of scanning transmission electron microscopy and energy-dispersive X-ray spectrometry using a windowless multiple detector, a high-brightness Schottky electron gun, and a spherical aberration corrector in the illumination probe optics. A small routine was developed to deconvolute the respective compositions of γ′ and γ″ nanoprecipitates embedded in the γ matrix. Keeping the electron probe current low enough—a few hundred pA—prevented excessive irradiation damage during the acquisition of element maps and brought their spatial resolution down to the atomic column level to track their element compositions. The present results agree with and complement atomic probe tomography observations and Thermo-Calc predictions from the literature. The presence of an Al enrichment at the γ′/γ″ interface—which may control the γ″ phase coarsening—is observed in the last row of Al-Nb-Ti columns along this interface. In addition, a few columns with similar composition changes are found randomly distributed in the γ′ phase.

## 1. Introduction

Several examples of EDXS analysis at atomic column resolution have already been published. Most of these have been obtained on oxides [1,2], nitrides, and carbide ceramics with a main accent on perovskites like SrTiO_3_ and semiconductors. But, to the authors’ best knowledge and with the exception of nanoparticles on a supporting film, such examples still remain scarce for ductile materials such as metals and alloys, which often have smaller lattice spacing, are most often prone to knock-on irradiation damage [3,4], and are more difficult to prepare as very thin foils frequently warp under the effect of internal stress relaxation or milling ion implantation.

Although Inconel 718 (IN718) is a well-known and widely used Ni-based superalloy, precise observation of the γ′ and γ″ particle morphology, as well as determination of chemical element partitioning between these two phases, remains problematic. Recent advances in the production of more intense, thinner electron probes and more efficient X-ray detectors narrow the gap between Energy-Dispersive X-ray Spectrometry (EDXS) and Electron Energy Loss Spectroscopy (EELS) [5]. As such, the ChemiSTEM™ technology [6] used in his work offers higher X-ray count rates and better quality elemental maps, which are essential features for distinguishing γ′ from γ″ nanoprecipitates, measuring their size, and determining elemental distributions.

The distribution, structure, and chemical composition of strengthening nanoprecipitates in nickel-based superalloys are still a matter of investigation. For instance, recently, Hosseini [7] and Guth [8] conducted extensive work on the mechanical properties and structure of additively manufactured IN718. Over the past twenty years, high-resolution electron microscopy (HRTEM) and atomic probe tomography (APT) have provided the best spatial resolution for structural and composition investigation. In a different approach, Lawitzki [9] has shown that SANS neutron data can derive accurate γ, γ′, and γ″ phase partition after deconvolution with TEM and APT support. Recent advances in Scanning Transmission Electron Microscopy (STEM) imaging using High-Angle Annular Dark Field (HAADF) detectors and in chemical nanoanalysis via energy-dispersive spectrometry with large X-ray collection efficiency [6] open new ways for distinguishing nanophases from each other as well as from their matrix. These new capabilities are applied in this work to IN718, made using conventional technology, where TEM and HRTEM failed [10,11]. Dark Field Transmission Electron Microscopy (DF-TEM) images distinguish the distribution, shape, and size of the single variant of γ″ precipitates alone or undiscriminated γ′ + γ″ precipitates together, but never the γ′ precipitate alone [12,13]. In principle, a difference image between two DF-TEM images taken with different diffraction reflections could bring the γ′ distribution after subtraction of the γ″ component. If this approach is useful in small areas, it fails in practice on areas large enough to measure volume phase partitioning due to the inevitable changes in dynamical intensities and excitation errors that always occur on slightly curved samples after strain relaxation during thinning.

In the present study, a novel approach to the structure of γ′ and γ″ nanoparticles as well as chemical element partitioning between the three phases down to the atomic level takes advantage of recent progress STEM imaging using HAADF Z-contrast, atomic resolution, and EDX spectrometry. However, most of the high spatial resolution EDXS analyses and atomic column resolution maps of thin films concern brittle materials such as ceramics and semiconductors. This can be due to the great interest in these materials for advanced technologies, but also to the relative ease of preparing suitable thin films via ion milling, mechanical polishing, cleaving, or Focused Ion Beam (FIB) cutting [14]. In comparison, the thinning of ductile materials, particularly metals and alloys, for high-resolution analysis or mapping with large areas for statistical relevance is more demanding. Very low-energy ion milling to remove surface oxide after electrochemical thinning, cold work of the surface after mechanical polishing, or surface irradiation of FIB foils are hindered by the buckling that arises from the high ductility and stress relaxation in this type of material. In addition, preventing excessive knock-on irradiation damage instead of radiolysis during observation would call for using lower acceleration voltage to operate below the atomic displacement threshold energy but at the cost of a reduced microscope performance.

Distinguishing between the three phases using the chemical “Z-contrast” in STEM-HAADF images works well on thin samples despite the proximity of the “effective atomic” numbers of the matrix and the γ′ phase. However, at thicknesses greater than a few tens of nanometers, the γ′ precipitates may be partially or even totally buried in the matrix, reducing their relative contrast. In addition, additional contrasts may appear as disturbances due to large-angle diffraction, the effect of channeling, and possible roughness of the sheet. Thus, EDXS-STEM is the only method of obtaining the distribution of the elements and therefore the partitioning of phases on large-area maps as well as on the nanometer scale, even down to the resolution of atomic columns. For Ni-based superalloys, this constitutes the only alternative to atomic probe tomography [15,16], which was previously used for the quantitative analysis of γ′ and γ″ nanoprecipitates and the composition of their interfaces. It is important to note here the complementary nature of atomic resolution observation capabilities, which are in-depth with APT and lateral with HRSTEM and EDXS.

The techniques we used are very general and can be applied to other materials as long they can withstand electron irradiation. But quantifying the composition of buried precipitates requires at least one chemical element to be present in only one phase and to write a quantification software beyond the usual ones. Therefore, in addition to the study of the structure of the In718 sample, the main objective of this work, didactic information and simulations, have been added to the interpretation process to draw the reader’s attention to the biases inherent in high-resolution EDXS analysis of buried structures.

## 2. Materials and Methods

**Figure 1:**
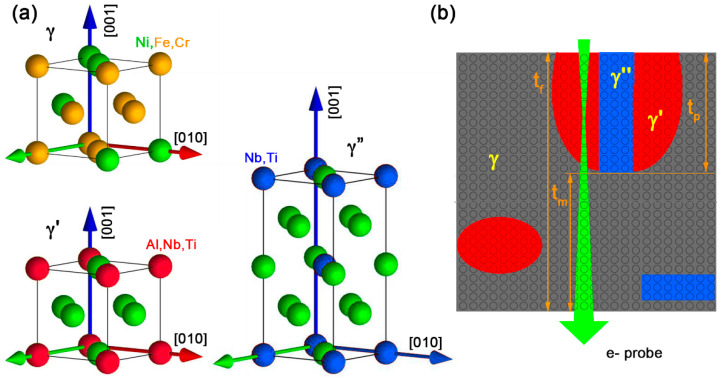
(**a**) Ball models of the γ matrix and the γ′ and γ″ phases around their [0,0,1] zone axis; (**b**) the contrast of the nanoprecipitates buried in the matrix (γ′ in red and γ″ in blue) only contribute to a fraction of the projected image. Moreover, the electron probe only remains in perfect focus in a thin slice of the foil.

The IN718 superalloy studied in this work has a nominal composition Ni–19Fe–18Cr–5Nb–3Mo–1Ti–0.5Al–0.04C (wt%) and received a standard heat treatment followed by ageing at 650 °C/48 h + 730 °C/4 h, air cooling. More details are provided in Kulawik [10,17]. The classical models for the γ, γ′, and γ″ phases are shown in Figure 1. The crystal data used for all the three phases latice simulations are shown in Table 1. They have been rounded to avoid mismatch and strain.

Thin foils for TEM/STEM investigations were prepared by mechanical pre-thinning down to 45 µm followed by electropolishing in a double jet (10% perchloric acid + glacial acetic solution) at +10 °C/50 V (Tenupol 5, Struers, Ballerup, Denmark). To increase the chances of observing grains in the [0,1,0] direction without tilting the sample holder more than a few degrees to keep the best EDXS and HRSTEM conditions, some thin lamellae were also prepared using FIB on suitable areas selected via Electron Backscatter Diffraction EBSD (HKL Channel.5, Oxford Instr., Abingdon Oxfordshire, UK) using a Scanning Electron Microscope SEM (XL30, FEI, Hillsboro, OR, USA).

Characterization of the IN718 strengthening phases was performed at 200 kV via HAADF-STEM, electron diffraction, and EDXS using an FEI Titan^3^ G2 60-300 analytical microscope (FEI, Hillsboro, OR, USA) fitted with a monochromator, a probe Cs corrector (DCOR, CEOS GMBH, Heidelberg, Germany), and the ChemiSTEM technology (X-FEG field-emission electron gun and four windowless Super-X EDXS detectors) [6,18]. In comparison with former analytical TEM designs, this technology provides higher EDXS-STEM spatial resolution, higher X-ray photon collection efficiency for faster acquisition of spectra and maps, thus, reduced irradiation damage for collecting the same EDXS counts.

The X-ray spectrometer Quantax SuperX running the Esprit 1.9 interpretation software 1.9 (Bruker Nano GmbH, Berlin, Germany) was used to gather EDXS Super-X data and first-level data processing. Further interpretation, spectra simulation, and evaluation of possible artifacts were carried out using ImageJ [19], NIST DTSA-II [20,21,22], MC X-ray Lite [23], Casino [24], and Mathematica (Wolfram Research Inc., Champaign, IL, USA). The interpretation and simulation of diffraction patterns, high-resolution HAADF-STEM images, and channeling/dechanneling effects were performed using Digital Micrograph 1.8 (Gatan Inc., Pleasanton, CA, USA) and Java Electron Microscopy Software (JEMS).

## 3. Results and Discussion

The TEM/STEM observation shows that the matrix of IN718 superalloy after a standard heat treatment contains disc-shaped γ″ and spheroidal γ′ precipitates, γ′/γ″ co-precipitates, and γ′/γ″/γ′ sandwich-like as well as some larger particles of the δ phase and primary niobium- and titanium-rich MC carbides and/or M(C, N) carbonitrides (M = Ti and/or Nb). The microstructure of the investigated IN718 is described in detail by Kulawik [10,17]. According to Geng et al.’s calculation [25] and Miller’s APT observation [26] an Al enrichment occurs along the γ′/γ″ interface and interface-controlled coarsening is likely a predominant kinetic process while the precipitates form co-precipitates to prevent a loss of γ″ coherency with the γ matrix [27].

### 3.1. HAADF-STEM Imaging

The atomic number contrast (*Z*-contrast) observed in the HAADF-STEM images should make it possible to differentiate between phases owing to its sensitivity to their specific atomic number *Z*. However, it should not be forgotten that the HAADF intensity at a location (*x*,*y*) depends not only on atomic number *Z* but also on the local sample thickness *t*(*x*,*y*) and possible changes in electron channeling induced by the presence of crystal defects.

In order to observe the distribution and nature of precipitates over a sufficiently large field of view for a statistical study, the preparation of TEM foils with a thickness *t_f_* of less than 50 nm remains a challenge in ductile materials due to warping induced by the relaxation of internal stresses. In practice, it implies that the spheroidal or rounded platelet nanoprecipitates observed in IN718, with characteristic dimensions 5 to 30 nm, are partly or even entirely buried in the matrix and only in a part contribute to the contrast observed at their location (Figure 1b). As a consequence, changes in the brightness in HAADF images can no longer be understood without a local measurement of the relative thickness of the precipitate and the matrix that surrounds it.

As a first approximation, HAADF imaging is an incoherent process, and the intensity *I*(*x*,*y*) at the *x*, *y* position results from the convolution of the electron probe wave function *C*(*x*,*y*) and the specimen’s projected potential *P*(*x*,*y*):

(1) $$I\left(x,y\right)={\left|C\left(x,y\right)\right|}^{2}⨂{\left|P\left(x,y\right)\right|}^{2}$$

At the quite large angles required for HAADF and neglecting the contribution of inelastic events, the projected potential peaks at the atomic nucleus depend on the Rutherford scattering cross-section proportional to the square of the specimen atomic number *Z*^2^. This exponent is reduced when the nucleus screening by bound electrons is taken into account and ranges from *ε* = 1.6 to 2.0 depending on the inner HAADF detector diameter [28,29,30,31]. Therefore, at low and intermediate magnification where the probe extends on distances larger than the interatomic distances in the unit cell, the intensity becomes proportional to the average of the projected potential squared:

(2) $$I\left(Z\right)\approx Z^{\epsilon }$$

Neglecting absorption and considering incoherent illumination, the intensity for a thin foil sample becomes proportional to its local thickness *t_f_*:

(3) $$I\left(Z\right)\approx Z^{\epsilon }t_{f}$$

In binary materials containing two atomic species, A and B, with atomic concentrations *c*_A_ and *c*_B_, respectively, the pure element atomic number *Z* is replaced by an effective atomic number *Z*_eff_, which is the mean square of individual phase contrasts balanced by their concentrations. It can be extended for n elements:

(4) $$Z_{\mathrm{eff}}^{\epsilon }=\sum_{1}^{n}c_{i}Z_{i}^{\epsilon }$$

The mean atomic number 〈*Z*〉 is often close to *Z*_eff_ for unfiltered energy intensities and used for quick image interpretation:

(5) $$\left\langle Z\right\rangle =\sum_{1}^{n}c_{i}Z_{i}$$

*Z* values of the γ, γ′, and γ″ phases according to their mean composition measured in this work are 27, 28, and 30, respectively, which suggest a noticeable HAADF contrast on high-quality maps. However, if we take into account the fact that the precipitates are buried in the matrix, their actual contrast *C* compared with the pure matrix is significantly reduced (Table 2):

(6) $$C=\frac{\left[Z_{p}^{2}t_{p}{+Z}_{m}^{2}\left(t_{f}-t_{p}\right)\right]}{Z_{m}^{2}t_{f}}$$

with thickness indices *t_p_*, *t_m_*, and *t_f_* holding for precipitate, matrix, and foil, respectively.

Therefore, HAADF-STEM contrast is insufficient to discriminate the three γ, γ′, and γ″ phases in foils in medium thickness (≈80 nm) of “difficult” materials such as Ni-based superalloys. More information is required for this aim and in this instance EDXS mapping provides additional chemical contrast while retaining the spatial resolution close to that of HAADF.

**Figure 2:**
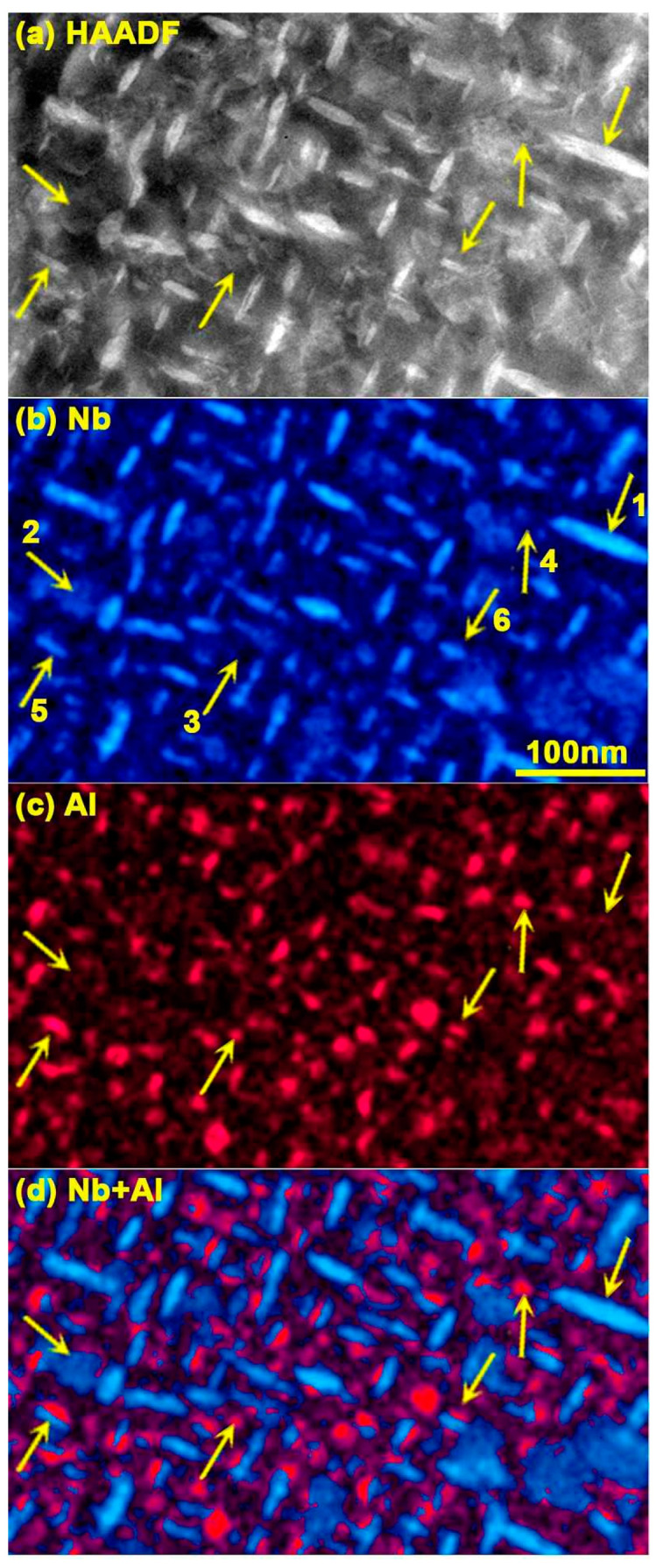
Comparison of the Nb and Al precipitate distributions (EDXS net counts): HAADF (**a**), Nb (**b**), Al (**c**), and sum Nb + Al (**d**). Arrows show γ″ disc precipitates viewed edge-on with strong contrast (arrow 1) and in plane view with weak contrast (arrow 2) in (**b**,**d**). Spheroidal or lenticular γ′ is present (arrows 3 and 4) with strong contrast in (**c**) and weak contrast in (**a**). Both γ″ in plane view and γ′ are hardly visible, if not at all, in HAADF on the matrix structure because of dislocation contrast (**a**). Arrows 5 and 6 point at a γ′/γ″ co-precipitate and a γ′/γ″/γ′ sandwich precipitate.

Figure 2 compares a HAADF image with its corresponding EDXS net count maps of Ni and Al recorded along [0,0,1]γ. The bright areas of the disc-shaped Nb-rich γ″ precipitates seen edge-on and elongated along [1,0,0]γ (arrow 1) or [0,1,0]γ directions are easily observable on the Nb map (Figure 2b). These discs, several tens of nanometers in diameter, are present in a large part of the foil thickness and also appear bright in the HAADF image (Figure 2a). The arrow (2) points to a γ″ disc seen in plane view. Its thickness is a fraction of that of the foil and its contrast is much weaker contrast in the EDXS Nb map. It cannot be clearly seen in the HAADF image and may be confused with defects or thickness changes in the matrix. The Al EDXS map (Figure 2c) shows numerous precipitates lying out of the γ″ ones or stuck along them, forming γ′/γ″ co-precipitates or γ′/γ″/γ′ sandwich-like particles. They correspond to spheroidal or lenticular γ′ precipitates. Most of them are not visible on the HAADF image because their effective atomic number is too close to that of the matrix and their thickness is too small compared with that of the foil (arrows 3 and 4).

**Figure 3:**
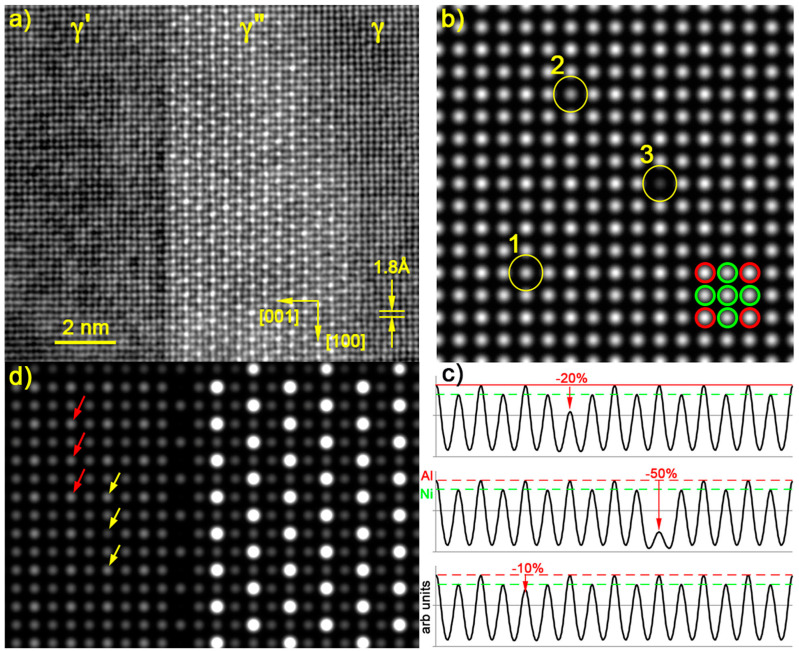
HAADF-HRSTEM image of an edge-on γ″ disc surrounded by a γ′ precipitate (**left**) and matrix (**right**). The γ′ pure Ni and Al-Nb-Ti columns and the γ Ni-Cr-Fe columns exhibit very similar contrast; Al-Nb-Ti “defective columns” are also present as dark areas along the γ′/γ″ interface as well as randomly dispersed in the γ′ phase (**a**). HAADF simulation of a 24 nm thick foil γ′ shows a very weak contrast between healthy pure Ni (green) and Al-Nb-Ti (red) columns. It contains three “defective columns” in which, respectively, 10% (1), 20% (2), and 50% (3) of the Nb atoms have been randomly replaced by Al atoms (**b**). Profiles along horizontal rows traveling alternately through pure Ni and perfect Al-Nb-Ti columns and crossing “defective columns” 1, 2, or 3, respectively (**c**). Simulation of a γ′/γ″ interface similar to that in (**a**), assuming a foil 68 nm thick and an exchange of all Nb for Al atoms in the rightmost γ′ Al-rich columns (interface). The median yellow arrow points to a defective column where all Nb was replaced by Al only in the uppermost third part of the column while the other two arrows point to healthy columns. Similarly, the red arrows correspond to a replacement in the lower third of the column only (**d**).

High-resolution STEM microscopes fitted with a spherical aberration Cs-corrector in the illumination (probe) section provide images of atomic columns with a resolution better than 0.1 nm (Figure 3a). They add useful information for phase identification via the crystal lattice structure though this reduces the observed field of view to one or two tens of nanometers on 2048 × 2048 pixel images. To achieve such a resolution, the probe size needs to be reduced below the distance between atomic columns and the convolution product in Equation (1) has to take into account the actual intensity probe shape in *C*(*x*,*y*). The actual probe size and profile change with thickness due to the geometrical broadening according to the illumination convergence angle, channeling/dechanneling effects, and atomic disordering due to phonons. Therefore, Equation (3) no longer holds if the foils are not very thin. The column images always appear bright and their intensity increases with *Z*. But when the focused probe penetrates deeper into the foil, defocusing, channeling, and cross-talk with neighboring columns occur and break the linear relationship between contrast and thickness *t_f_* [32]. The atomic resolution of periodic structures is maintained in quite thick samples—a hundred nanometers or so—but cross-talk with adjacent columns occurs through channeling/dechanneling and phonon scattering and HAADF contrasts no longer follow the simple atomic number scheme. This effect is now widely documented; for instance, in [33].

Consequently, a full understanding of HAADF-HRSTEM intensities and image contrast requires image simulation including the sample’s crystal structure, its thermal dynamics (phonons), foil thickness, probe/sample orientation, and probe-forming optics. The comprehensive program JEMS for (HR)TEM and (HR)STEM imaging and diffraction was used in this work [34]. The reader will find an extensive list of references to other software—some of which are freeware—in [35] to which DrProbe [36,37] and MULTEM [38,39] may be added, for instance.

Figure 3a shows a [0,1,0] HAADF-HRSTEM image of a part of a γ′/γ″/γ sandwich viewed edge-on. The brighter contrast of the Nb-rich columns in g” with respect to that of the adjacent Ni ones is obvious. But the one between the Al-Nb-Ti and pure Ni columns in the γ′ phase is barely visible, seems unreliable and is certainly too weak in the usual images from samples around 50 nm thick, recorded at an economical electron dose to avoid irradiation damage (exposure time of around 10 s for a 512 × 512 pixel image with a 200 kV probe, a current of 200 pA and a semi-convergence angle of 16 mrad). So-called defective Al-Nb-Ti columns show dark contrast along the γ′/γ″ interface. Some of these are also randomly present in the γ′ precipitate body, but mostly with a reduced contrast change. These “defective columns” were already present at the beginning of observation and even with less intense electron probes than those used for EDXS mapping.

As a guide, the *Z*^2^ of [0,1,0] atomic columns is summarized in Table 3 with respect to their specific compositions. The Nb-Ti columns have a much larger *Z*^2^ than the pure Ni ones in the γ″ phase and are easily distinguishable in Figure 3a. But the difference in *Z*^2^ for Al-Nb-Ti columns compared with the pure Ni ones in the γ′ phase is much smaller—it even reverses when considering <*Z*> or *Z*_eff_—and in practice, the only striking contrast change between the **γ**’ and **γ** phases is the noisier lattice in the **γ**’ phase.

For a more accurate interpretation, an HAADF-HRSTEM image simulation was performed with the multislice method using the JEMS software [34]. In this example, a 24 nm thick [0,1,0] foil with a random partial occupancy of the Al-Nb-Ti sites was considered, keeping on average the γ′ phase composition from Table 3 (Figure 3b,c). A supercell made of 8 × 8 γ′ unit cells contains three “defective columns” (numbered 1, 2, and 3) where 10%, 20%, and 50% of the Nb atoms, respectively, were randomly replaced by Al ones that correspond to an exchange of 2, 5, and 11 atoms out the 64 atoms contained in a column. These “defective columns” show contrasts similar to those observed in Figure 3a, although the replacement of only two Nb atoms in column (1) is barely visible. Figure 3d is a simulation of the γ′/γ″ interface for a 63 nm thick foil (176 atoms in total in a γ′ column). All Nb atoms were replaced by Al atoms in the last γ′ row along the interface and the corresponding contrast became too low to be visible with the dynamic range in this picture. In addition, two “defective columns” were created inside the γ′ body. In each set of three arrows of the same color, those at the top and bottom point to healthy columns for reference. The median arrow points to a defective column where a third of the Nb atoms (21 out of 65) were replaced immediately either below the entrance face of the foil (yellow arrow) or above the exit face (red arrow). The change in contrast is greater when the upper atoms are replaced rather than the lower ones. This difference is attributed to the increasing broadening of the beam with depth in this rather thick foil and possible crosstalk with adjacent columns. It is worth noting on the one hand that this difference in contrast, which is detrimental to phase identification, is useful for determining the depth of nanoprecipitates embedded in foils; for instance, in [40]. But on the other hand, the apparition of crosstalk may introduce a significant bias in EDXS during quantitative analysis and apparent shifts in atomic positions at the level of atomic column resolution [41,42].

### 3.2. Energy-Dispersive X-ray Spectrometry

**Figure 4:**
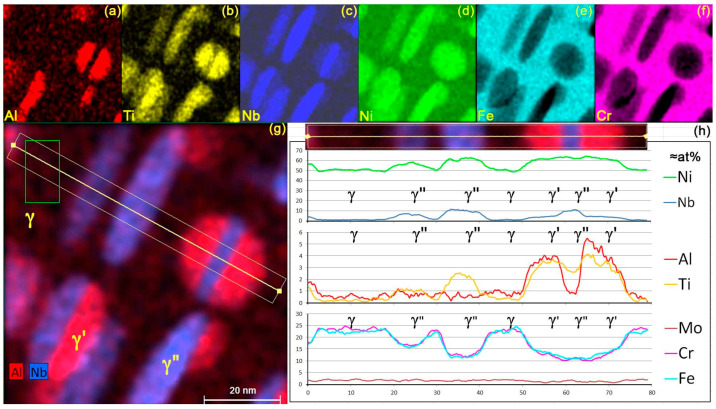
“Pseudo at%” EDXS maps (**a**–**f**). Composite Al/Nb map with area (green rectangle) for pure matrix composition measurement and position of the linescan across matrix (γ), γ″ and complex γ′/γ″/γ′ precipitates (**g**). “Pseudo at%” composition profiles (**h**).

**Figure 5:**
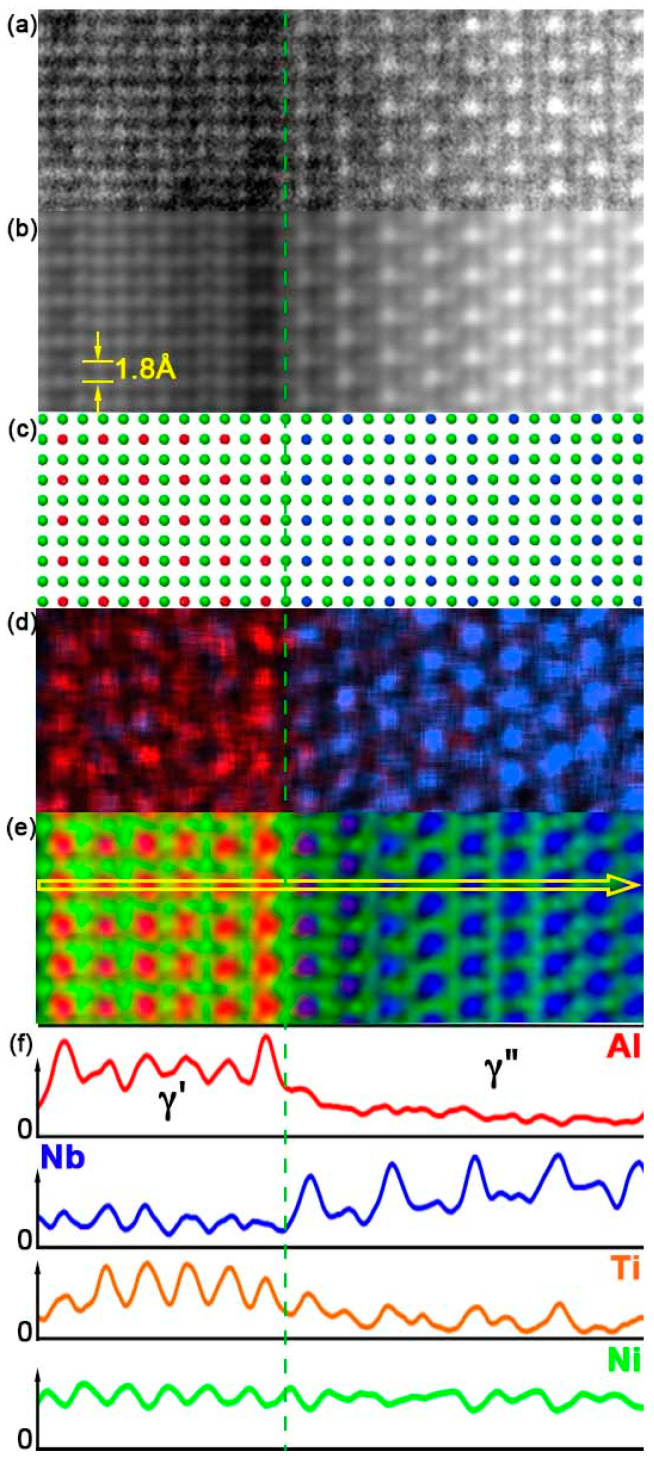
Analysis of a γ′/γ″ interface seen along [0,1,0]γ″. Original HAADF image with several defective (darker) unit cells (**a**). The same area is averaged by iteratively adding lattice cells in the (vertical) direction parallel to the interface (**b**). Ball model: pure Ni (green) columns, Al-Nb-Ti (red) columns in the γ′ phase, and Nb-Ti (blue) columns in the γ″ phase (**c**). Composite Al/Nb EDXS raw counts map (**d**). Al-Nb-Ni composite EDXS raw count map smoothed with a Gaussian filter (**e**). Original Al-Nb-Ni composite EDXS raw count map averaged along vertical rows (**f**). Element profiles (arbitrary units), corresponding to the yellow arrow in (**f**), across the interface of rows containing alternate Al-rich and pure Ni columns Strong Al enrichment, Nb, and Ti depletion are observed on the γ′ defective unit cell row along the interface as well as on the left-most row (**g**).

Progress in EDXS analysis efficiency has brought this technique to the level of element mapping and quantification to the nanometer level (Figure 4) and even to the level of atomic column resolution (Figure 5). It has the advantage of greater tolerance on sample thickness and smaller ionization delocalization than in electron energy loss spectroscopy but at the expense of a less efficient signal collection, i.e., the need to use a higher electron dose that increases the risk of electron irradiation damage.

For instance, the Super-X EDXS detector used on the Titan^3^ G2 60-300 analytical microscope has a set of four 30 mm^2^ windowless SDD diodes arranged symmetrically around the sample, which increases the collection angle from 0.13 sr in older 10 mm^2^ detectors to ≈ 0.7 sr as long as samples remain normal to the probe, i.e., a gain of ≈ five times [6,43]. Furthermore, the absence of a front window at the entrance detector reduces X-ray absorption and increases sensitivity for low-energy X-ray photons extending the EDXS efficiency toward light element analysis, a field hitherto reserved for electron energy loss spectroscopy. In addition, a high-brightness Schottky X-FEG gun and a DCOR spherical aberration corrector in the probe-forming optics maintain resolution better than 0.2 nm, even with probe currents as high as 2 nA at 300 kV, in probes with a low convergence angle.

In practice, the new limitations of EDXS analysis arise from the balance between the ability of thin foils to withstand high current density and the dose of electrons needed to obtain a sufficient number of X-ray photons for statistical relevancy, and from the broadening of the probe and channeling/dechanneling when traveling through samples thicker than one or two tens of nanometers (see, for instance, Lu on SrTiO_3_ perovskite [44]).

Nowadays, acquiring qualitative EDXS mapping at atomic column resolution is easy enough with high-level analytical microscopes. But quantitative analysis column by column remains a challenge, requiring accurate consideration of probe scattering across the thin foil as a convolution of three terms: (i) the geometric broadening of the probe, which is easily calculated at any depth in the sample; (ii) the oscillation of the electron density in the probe along its primary direction due to channeling/dechanneling effects at increasing depth, which depends on the composition of the column under examination; for instance, ALCHEMI in [45]; (iii) the thermal disorder of atom positions (phonons); and (iv) the dependence of the ionization cross-section on the distance between the incident electron and the center of the target atom to account for interaction delocalization (impact parameter) [46,47,48], which is generally not precisely known. Therefore, the quantitative analysis is still a matter of tough development [49,50,51,52] and is nowadays at best only a semi-quantitative analysis that research groups have to develop themselves, often with simplifying assumptions (Gaussian ionization cross-sections, given sample thickness, set of possible column composition for channeling estimation, no electron absorption, etc.). For instance, in [44,53,54] and references herein.

Another aspect of EDXS spectrometry that is all too often neglected is the implicit assumption made in most softwares used today of a constant composition throughout the entire probe–sample interaction volume, including the X-ray output path to the detector. In particular, this excludes multilayer foils or the presence of a second phase in a matrix. In these latter cases, recording a “hybrid” spectrum containing the sum of the X-rays emitted by each phase makes it impossible to distinguish their origin and to determine the composition of each phase. In favorable cases, the matrix contains one or more elements that are absent in the second phase. Using Esprit, weighted subtraction of a spectrum recorded on the matrix alone from a hybrid matrix-plus-second-phase spectrum until the characteristic element of the matrix disappears results in a spectrum of the second phase alone that can now be quantified.

When foils larger than a few tens of nanometers, these concentrations require correction for the bias introduced by the absorption of generated X-ray photons as they exit toward the detector. Phase thickness is easily deduced if an element has well-distinct K and L (or L and M) lines. As these are absorbed differently, the ratio of their intensities measured on a pure matrix region leads directly to the foil thickness. Extending the process on the hybrid spectra leads to the thickness of the second phase. However, to achieve an accurate correction, it would still be necessary to determine the height of the second phase and the nature of the absorber—second phase and/or matrix—encountered between the X-ray photon generation point and the surface. This step has not been taken into account in this work and is still the subject of a development to be published on biases in quantification using the transmission microscope.

As an alternative to subtracting spectra that suffer from rather poor statistical relevance and thickness changes in precipitates, a routine was written in ImageJ applied to raw maps to create single phase maps by subtracting the matrix, present throughout, with a floating weighting. These single element maps were then quantified with Esprit and the results are presented in Table 4 below.

#### 3.2.1. Composition of the γ′ and γ″ Phases

Knock-on damages IN718 superalloy too rapidly under a 300 kV electron probe to obtain high-quality EDXS concentration maps at intermediate magnification or even atomic column maps in HRSTEM. Reducing the accelerating voltage to 200 kV brings a significant improvement, although it still remains above the irradiation threshold. The 200 keV electron dose was kept to 1 × 10^−10^ Cb/nm^2^ (430 pA, 20 min acquisition time) to stay below the limit of visible irradiation damage. Esprit software was used to interpret the collected “data cube” (EDXS spectra for each pixel of the map) to produce individual element maps and linescans. Because Ni is not only present in the matrix but also in the γ′ and γ″ precipitates, the popular method of retaining only the elements present in the precipitate for quantitative analysis fails using the built-in software.

Figure 4a–f shows the “pseudo at%” elemental maps, without absorption correction, for each main chemical element present in IN718 (a–f), a composite map where Al and Nb are superimposed (g) with the linescan trace (80 nm × 6.4 nm) used to track the hybrid element concentration across the γ matrix and the γ′ and γ″ phases (h). The foil orientation as been slightly shifted away from [0,1,0] to minimize electron channeling effects while keeping the γ′/γ″/γ′ interfaces of the sandwich-like precipitates close to the incident beam direction.

The γ matrix atomic composition measured in the green rectangle and obtained with Esprit standardless quantification is provided in Table 4, “EDXS Esprit matrix only γ ± 2σ” with a 95% statistical relevancy but without absorption correction. The ratio of the intensities between the Ni-K and Ni-L lines corresponds to a 130 nm foil thickness. The element profiles on the line scan confirm that Cr, Fe, and Mo are essentially present in the matrix, while Nb and Ti belong to precipitates.

The situation becomes more complex for areas containing buried γ′ or γ″ phases. In all cases, the comparison of the Cr and Fe profiles with those of Al, Ti, and Nb shows that they often correspond to a sandwich-like γ′/γ″/γ′ precipitate buried in the matrix and confirms that the γ″ phase is richer in Nb and poorer in Al than the γ′ phase. The progressive change and the element content show that γ′ precipitates have a hemispheroidal or lenticular shape and may stick to both sides of a γ″ disc. Although several models of structures consider Nb to belong only to the γ″ phase, the profiles in Figure 4h show that it is also significantly present in the γ′ phase. Traces of aluminum might also be present in the γ″ phase. The low Cr and Fe contents in the large edge-on γ″ precipitates as well as their progressive concentration change from the matrix to the γ′ phase or the in-plane γ″ phase show that nanoprecipitates contain little or none of these two elements, confirming the atomic probe tomography (APT) observations of Miller [16,26] and Geng [25] on similar IN718 alloys.

To obtain the composition of the buried precipitates, the matrix contribution is removed from the γ′ and γ″ hybrid spectra by subtracting a weighted amount of its spectrum. However, this approach yielded unreliable results possibly because of the poor statistical relevancy of spectra gathered over small areas and the complex shape of precipitates. Eventually, more robust results were obtained from 2.5 nm/pixel maps instead of spectra, after averaging the 600 × 600 pixel hybrid map with 8 × 8 binning, removing the background and building a stack of individual element maps. A small routine written in ImageJ is used to subtract the matrix component with a running weight to remove Cr and Fe from each pixel. Thresholding is then preferentially applied to the Nb histograms to separate areas containing the γ′ phases from the γ″ phases. Lastly, precipitate concentrations and actual concentration maps are calculated using the Cliff–Lorimer quantification method, bearing in mind that the best accuracy is obtained in areas where matrix and precipitates occupy a significant part of the foil thickness. These deconvoluted at% concentrations were averaged over the whole map area and have a higher statistical relevancy. The reported uncertainties represent the standard error between several analyses while changing the criteria of Cr or Fe absence in the precipitates, the Nb thresholding level, and the size of the rejected boundary around precipitates where thickness becomes too low for an accurate estimate of Ni content. These results for γ, γ′ and γ″ phases are reported in Table 4 as “EDXS ImageJ deconv.”. Comparison with the matrix composition obtained using ESPRIT quantification in the 100 nm^2^ green rectangular pure matrix area in Figure 4h (statistical relevancy 2σ) confirms that the main uncertainty on compositions may stem from the Cliff–Lorimer coefficients, the limited number of counts for low concentration elements, and the neglect of the absorption correction for Al, but not from the ImageJ deconvolution procedure itself.

These results are close to the compositions obtained via atomic probe tomography (APT) and Thermo-Calc simulation [16] on an IN718 alloy with close composition but with slightly different heat treatment. The high Nb content in the γ′ phase is confirmed. The presence of about 1.5 at% of Al in the γ″ phase was not expected from APT and Thermo-Calc values, but simulations with the DTSA-II Halley software [21] exclude an artifact due to scattered electrons hitting the thick part of the foil after spiraling in the magnetic field of the objective pole pieces [54] or an inaccurate background subtraction. However, this value could be overestimated due to the impossibility of performing a thickness correction for X-ray absorption on Al in the absence of information on the height position of the precipitate in the foil thickness.

The nickel content in the pure matrix is determined using the EDXS ESPRIT quantification software or that obtained by the EDXS-STEM map deconvolution are in good agreement with each other. A longer counting time or a richer map should be considered to improve statistical relevance on low-concentration elements. This good overall match, obtained with the assumption “no Cr” as well as “no Fe”, confirms the negligible Cr and Fe contents in precipitates. The lower Ni value in the APT results may be due to differences in the overall composition of the alloy, in particular its lower Ni content and the presence of Co, or to a different heat treatment. Table 3 compares the EDXS results obtained in this work with Miller’s APT results [26] and with his Thermo-Calc simulation [16]. The Al/Ti ratios measured via EDXS match the calculated ratios in the γ′ phase and in the matrix better than those from APT, but reflect the higher Al content in the γ″ phase. The Ti/Nb ratio follows the opposite trend with a better match in the γ′ phase and the matrix for APT but a large overestimation in the γ″ phase, whereas EDXS is the best. However one should keep in mind that samples have slightly different compositions and heat treatments.

#### 3.2.2. Chemical Information at Interfaces and Defects

Crystal structure information gathered from HAADF-STEM images can be extended to EDXS-HRSTEM chemical information with atomic column lateral resolution. This complements the unrivaled depth resolution of the APT investigation with lateral resolution and paves the way for structural analysis of edge-on interfaces and defects.

Probes thinner than the distance between atomic columns but still intense enough to produce sufficiently statistically relevant X-ray counts are nowadays available with modern probe-forming electron optics based on high-brightness electron guns and spherical aberration correctors. On the other hand, the sample must be thin enough to avoid crosstalk between columns due to the short depth of focus of Cs-corrected probes leading to enhanced geometrical broadening, electron inelastic scattering, and channeling/dechanneling along atomic columns. To compensate for it, lowering the sample thickness to ten or a very few tens of nanometers reduces the emitted X-ray intensity and requires compensating with a higher probe current or a longer acquisition time, which quickly leads to excessive irradiation damage and the occurrence of sample drift. On this last point, it should be noted that with a periodic lattice, drift correction systems often make mistakes during map acquisition by applying the drift correction to a neighboring column instead of the original column, which ultimately leads to inter-column mixing and concentration bias. This demonstrates the difficult balance of conflicting conditions between probe intensity and acquisition time (for instance, in [55] and related references). The recent progress of EDXS detector designs with improved X-ray collection efficiency is a breakthrough that brings EDXS analysis closer to EELS and APT techniques for high-resolution composition analysis and atomic site occupancy determination.

Part of the γ′/γ″ area in Figure 3a was observed at higher magnification for EDXS mapping at atomic column resolution close to their interface. The HAADF image shows Al-Nb-Ti “defective columns” in the γ′ phase all along the interface and several in its leftmost vertical row (Figure 5a). Conditions were chosen to prevent Moiré between the scanning raster and the crystal lattice and to keep irradiation low (256 × 256 pixels, pixel size 22 pm, probe convergence and intensity 16 mrad and 220 pA, respectively, acquisition time 159 s, total dose ≈ 3 × 10^−9^ Cb/nm^2^). This low dose leads to maps where the contrast of individual columns is quite noisy. Figure 5b is an average HAADF image obtained by summing the intensities of Figure 5a through iterative translations of one lattice period (0.35 nm) in the direction parallel to the interface, assuming that the structure is homogeneous along this direction. The structure of the interface becomes more visible, in particular, how horizontal γ′ and γ″ rows meet at the interface through a vertical row of defective Al-Ni-Ti columns. The leftmost presence of scattered defective columns also appears more clearly as vertical row but with reduced contrast. The ball model in Figure 5c corresponds to the atomic positions in Figure 5b.

The same process was applied to the original EDXS raw count maps. The large statistical noise on raw counts, due to low counts per pixel, prevents the calculation of net counts for each pixel (Figure 5d). A Gaussian blur by 3 pixels better reveals the column positions, but still with a large amount of noise (Figure 5e). Finally, the same iterative summing in the direction parallel to the interface is applied to equivalent horizontal rows (Figure 5f). It shows that:

(i) The γ′/γ″ phases join together via Ni-rich columns at the interface;
(ii) The γ′ Al-rich columns face the closest γ″ Nb-rich columns, a structure similar to “Geng model Figure 7a [25]” except that Ti comes back in the Al-Nb-rich γ′ columns in a proportion close to 33 at% for each of the three elements;
(iii) The presence of brighter Al-rich columns along the interface confirms the Al enrichment expected from ab initio calculations, Thermo-Calc simulations, and APT observation and that it extends in a single atomic plane only;
(iv) The “defective columns” in the leftmost Al-rich row show a similar tendency to compositional changes caused by the random replacement of Nb atoms by Al.

The yellow horizontal line scan in Figure 5f alternates between Al and Ni-rich columns in γ′, and then between Nb-Ti and Ni columns in γ″. The at% pseudo-concentration profiles are shown in Figure 5g. The Al content jump (Table 5) observed on different maps between the last healthy column before the interface and the interfacial column is relatively well reproducible, whereas those for Nb and Ti show notable fluctuations between them. This suggests at% compositions in γ′ for the last healthy and interfacial columns close to 31Al-35Nb-35Ti and 39Al-31Nb-30Ti, respectively. The apparent change in pseudo at% Ni composition between the Al-rich and Ni-rich columns in γ′ is of the order of 10% although Ni is not expected in Al-rich columns, or at least not in such large amounts. Similarly, Al is not expected to be present in Ni-rich columns. The presence of the small amount of Cr and Fe in the γ′ and γ″ phases suggests that these phases are buried in a gamma matrix, which is plausible given that the foil used was 105 nm thick. Using the previously estimated Cr/Ni and Fe/Ni ratios, the Ni contribution from the matrix was removed, but this process was not sufficient to remove all the Ni from the Al-rich columns. The original Esprit data cube shows that some Ni and Al are present throughout the map as a continuous background or the effect of cross-talk that cannot be removed due to the low number of counts collected to minimize the risk of irradiation bias. This limits the relevance of the column composition estimate.

## 4. Conclusions

Analyses of γ′ and γ″ precipitates in the IN718 superalloy were performed using STEM microscopy. An acceleration voltage of 200 kV was chosen to achieve the right balance between damage creation via electron irradiation, high-resolution HAADF-STEM imaging, and EDXS mapping with a probe current high enough to acquire EDXS data in one minute for atomic resolution or up to half an hour for quantitative analysis over 100 × 100 nanometer areas.

The γ″ precipitates are disc-shaped, with diameters of around 20 to 80 nm and thicknesses of 5 to 15 nm, about 20–80 nm in diameter and 5–15 nm thick, depending on the particular alloy heat treatment for the set of observed samples in this work. Some are sandwiched between hemispheroidal γ′ precipitates, forming so-called “sandwich-like” particles. Randomly distributed γ′ spheroidal precipitates of 8–25 nm in diameter are also present in the γ matrix.

The effective atomic number *Z*_eff_ of the γ″ phase is significantly larger than that of the γ matrix. At intermediate magnification, the γ″ discs are easily observed on HAADF images when they occupy a substantial part of the foil thickness, i.e., when viewed edge-on. On the other hand, in plane view, their thinness reduces their contrast to the level of the background noise in the matrix, making them barely visible. The *Z*_eff_ difference between the matrix and the γ′ phase is much smaller, and small spheroidal precipitates cannot be distinguished from the background noise when defects, such as dislocations, are present or damaged due to ion irradiation during thin TEM foil preparation. Consequently, HAADF-STEM is not sufficient to provide reliable phase distribution maps for precipitates in the tens of nm range and must be complemented by EDXS observation.

EDXS elemental mapping at intermediate magnification makes it easy to see the distribution of both γ′ and γ″ precipitates by observing Al and Nb net counts (or atomic “pseudo-compositions”). Interpretation of the intensity profiles confirms their shape. Assuming they contain negligible amounts of Cr and Fe, the compositions of the matrix and buried precipitates were deconvoluted using a routine written with the ImageJ software. In particular, this shows that Al and Nb in γ′ fcc Ni_3_Al and γ″ bct Ni_3_Nb models, respectively, are in fact replaced by 33Al-37Nb-30Ti at% and 6Al-78Nb-16Ti at%, neglecting Mo, whose concentration (close to 1%) is too low for a reliable estimation. These values are close to Miller’s observation [16] via APT and his Thermo-Calc simulations on an IN718 alloy, in which the composition and heat treatment were, however, slightly different.

HAADF-HRSTEM observation along [0,1,0]γ″ shows very bright Nb-rich atomic columns in γ″ precipitates compared with adjacent Ni-rich ones, making it easier to differentiate them from the γ matrix. On the other hand, the contrast between the Al-rich columns in γ′ precipitates and Ni-rich columns is low and this phase can barely be distinguished from the matrix. However, HAADF images reveal that γ′ precipitates may also contain darker columns, mainly observed along the γ′/γ″ interfaces, although some are also randomly distributed throughout the precipitate volume. Such “defective columns” appear to be richer in Al, which is compensated for by a deficiency in Nb and Ti. This suggests a smaller *Z*_eff_ and simulation of γ′ precipitates containing one “defective Al-rich column” among healthy ones show a similar contrast when some 20% of the Nb atoms are replaced by Al atoms. High-resolution EDXS maps show that:

- Al, Ti, and Nb in the γ′ phase occupy the corners of the unit cell cube of the ordered fcc crystal structure and Ni is at the face center;
- Compared with the Geng simulations, Ti is back in the γ′ Al-Nb-rich columns;
- Nb and Ti occupy the corner and center of the γ″ bct tetragonal lattice, with possible traces of Al;
- The γ′ and γ″ phases share a pure Ni atomic layer at the interface in γ′/γ″ co-precipitates and γ′/γ″/γ′ sandwich-like particles;
- The γ′ Al-rich columns face the nearest γ″ Nb-rich ones across the interface with a single Ni column in between, as calculations suggest;
- Darker columns of Al-Nb-Ti are observed in the HAADF images in the last γ′ row along the interface, suggesting an increase in Al and a loss of a part of heavier Nb and Ti elements. The EDXS maps at atomic column resolution show that they correspond to an Al enrichment that confirms calculation and APT observation. This corresponds to a loss of Nb and Ti but in an unclear proportion. The Ni columns at the interface remain unaffected;
- In addition, a few dispersed “defective columns” showing darker HAADF contrast and EDXS Al enrichment/Nb+Ti loss are randomly distributed in the γ′ phase.

These results demonstrate the complementarity nature of atomic probe tomography and scanning transmission electron microscopy. In particular, the former’s ability to carry out trace elements analysis and the latter’s ability to perform atomic column resolution investigations and microanalysis over areas large enough to be statistically relevant. In addition, it is essential to stress the complementarity of their resolutions, in-depth or lateral, which remain an essential element in the selection of the preferred investigation method.

## Figures and Tables

**Table 1:** Crystal data used for HRSTEM image simulations of γ, γ′ and γ″ phases.

Phase: Columns		Lattice	a	b	c
γ: 53Ni 24Cr 23Fe	Cubic	F m 3 m	0.365	0.365	0.365
γ′: 8Al 9Nb 8Ti/Ni	Cubic FCC	P m −3 m	0.365	0.365	0.365
γ″: 3Al 39 Nb 8Ti/Ni	Tetragonal BC	I 4/m m m	0.365116-117	0.365	0.73

**Table 2:** Expected relative HAADF contrast C between precipitates and matrix for the γ, γ′, and γ″ phases considering either 〈*Z*〉 or Z_eff_ atomic numbers.

Contrast in 80 nm Thick Foil	Phase	$C_{\left\langle Z_{\mathrm{eff}}\right\rangle }$	$C_{\left\langle Z\right\rangle }$	$Z_{\mathrm{eff}}^{2}$	${\left\langle Z\right\rangle }^{2}$
γ′ 20 nm diameter sphere, in the middle of the foil	γ	3.0%	1.8%	709	706
γ″ disc, edge-on view, extending through the whole foil thickness	γ′	32%	28%	794	757
γ″ disc in plane view, 5 nm thick	γ″	2.0%	1.8%	937	904

**Table 3:** Atomic numbers 〈*Z*〉 and *Z*_eff_ for atomic columns seen along [0,1,0] assuming the phase composition measured in this work (neglecting minor elements).

	Matrix γ	γ′	γ′ and γ″	γ″
[0,1,0] column composition	Ni_0.53_Cr_0.24_Fe_0.23_	Al_0.33_Ti_0.30_Nb_0.37_	Pure Ni	Nb_0.83_Ti_0.17_
〈*Z*〉//*Z*_eff_	26.6//26.6	26.1//28.7	28.0//28	37.8//38.4
Intensity ≈ 〈*Z*〉^2^//$Z_{\mathrm{eff}}^{2}$	706//709	679//823	784//784	1426//1477

**Table 4:** Atomic % composition: nominal alloy studied; concentration quantified with Esprit in a zone containing only the γ matrix; and concentrations deconvoluted with ImageJ in zones containing a buried γ′ or γ″ precipitate from Figure 4. Concentrations provided via APT and Thermo-Calc simulation (Table 1 and Table 3 in [12]).

	Al	Cr	Fe	Mo	Nb	Ni	Ti	Co	Al/Ti	Al/Nb	Ti/Nb
Nominal investigated alloy	1.1	20.1	19.8	1.8	3.1	52.9	1.2	-			
EDXS Esprit matrix only γ ± 2σ	0.5 ± 0.08	23.4 ± 0.52	22.4 ± 0.50	1.9 ± 0.07	1.0 ± 0.07	50.7 ± 1.06	0.1 ± 0.04	-			
EDXS ImageJ deconv. γ	0.6 ± 0.01	23.1 ± 0.18	22.3 ± 0.07	2.2 ± 0.01	1.00 ± 0.04	50.5 ± 0.10	0.3 ± 0.01	-	2.29	0.6	0.26
EDXS ImageJ deconv. γ′	7.2 ± 0.18	-	-	1.0 ± 0.12	7.9 ± 0.23	77.6 ± 0.53	6.4 ± 0.16	-	1.12	0.91	0.81
EDXS ImageJ deconv. γ″	1.5± 0.16	-	-	1.4 ± 0.07	19.6 ± 0.49	75.6 ± 0.28	4.0 ± 0.09	-	0.36	0.07	0.21
Nominal Miller’s alloy	1.27	19.6	21.5	1.76	3.24	51.15	1.18	0.31			
APT γ	0.58	23.48	25.54	2.13	1.59	45.83	0.47	0.38	1.23	0.36	0.3
APT γ′	9.11	0.79	2	0.53	7.22	72.88	7.38	0.09	1.23	1.26	1.02
APT γ″	0.35	2.16	2.11	1.3	20.07	68.37	5.59	0.05	0.06	0.02	0.28
Thermo-Calc γ	0.38	23.75	26.45	1.82	0.46	46.66	0.14	0.34	2.71	0.83	0.3
Thermo-Calc γ′	8.43	0.8	3.64	0.18	7.52	71.67	7.59	0.17	1.11	1.12	1.01
Thermo-Calc γ″	0.2	0.56	1.09	0.5	20.41	73.14	3.89	0.21	0.05	0.01	0.19

**Table 5:** Al-Nb-Ti column at% composition.

Column	Al	Nb	Ti
Interface	0.38	0.36	0.26
Last before interface	0.32	0.36	0.32

## Data Availability

Data are available from the authors upon reasonable request.

## References

[1] Batuk D., Batuk M., Abakumov A.M., Hadermann J. (2015). Synergy between Transmission Electron Microscopy and Powder Diffraction: Application to Modulated Structures. Acta Crystallogr. Sect. B-Struct. Sci. Cryst. Eng. Mater..

[2] Kwon J.H., Lu P., Hoffman J., Yuan R.L., Yoon A., Bhattacharya A., Zuo J.M. (2017). Elemental and Lattice-Parameter Mapping of Binary Oxide Superlattices of (LaNiO_3_)(_4_)/(LaMnO_3_)(_2_) at Atomic Resolution. Semicond. Sci. Technol..

[3] Egerton R.F. (2012). Mechanisms of Radiation Damage in Beam-Sensitive Specimens, for TEM Accelerating Voltages between 10 and 300 kV. Microsc. Res. Tech..

[4] Mansfield J.F., Okamoto P.R., Rehn L.E., Zaluzec N.J. (1987). Radiation Effects on X-ray-Microanalysis of a Light-Element Alloy in a Medium-Voltage Electron Microscope. Ultramicroscopy.

[5] Wenner S., Jones L., Marioara C.D., Holmestad R. (2017). Atomic-Resolution Chemical Mapping of Ordered Precipitates in Al Alloys Using Energy-Dispersive X-ray Spectroscopy. Micron.

[6] Schlossmacher P., Klenov D.O., Freitag B., von Harrach H.S., Steinbach A. (2010). Nanoscale Chemical Compositional Analysis with an Innovative S/TEM-EDX System. Microsc. Anal..

[7] Hosseini E., Popovich V.A. (2019). A Review of Mechanical Properties of Additively Manufactured Inconel 718. Addit. Manuf..

[8] Guth S., Babinský T., Antusch S., Klein A., Kuntz D., Šulák I. (2023). Creep–Fatigue Interaction of Inconel 718 Manufactured by Electron Beam Melting. Adv. Eng. Mater..

[9] Lawitzki R., Hassan S., Karge L., Wagner J., Wang D., von Kobylinski J., Krempaszky C., Hofmann M., Gilles R., Schmitz G. (2019). Differentiation of γ′- and γ″-Precipitates in Inconel 718 by a Complementary Study with Small-Angle Neutron Scattering and Analytical Microscopy. Acta Mater..

[10] Kulawik K., Buffat P.A., Kruk A., Wusatowska-Sarnek A.M., Czyrska-Filemonowicz A. (2015). Imaging and Characterization of γ′ and γ″ Nanoparticles in Inconel 718 by EDX Elemental Mapping and FIB-SEM Tomography. Mater. Charact..

[11] Phillips P.J., McAllister D., Gao Y., Lv D., Williams R.E.A., Peterson B., Wang Y., Mills M.J. (2012). Nano γ′/γ′’ Composite Precipitates in Alloy 718. Appl. Phys. Lett..

[12] Paulonis D.F., Oblak J.M., Duvall D.S. (1969). Precipitation in Nickel-Base Akkoy 718. ASM (Am. Soc. Met.) Trans. Quart..

[13] Sundararaman M., Mukhopadhyay P., Banerjee S. (1992). Some Aspects of the Precipitation of Metastable Intermetallic Phases in Inconel-718. Metall. Trans. A-Phys. Metall. Mater. Sci..

[14] Ayache J. (2010). Sample Preparation Handbook for Transmission Electron Microscopy.

[15] Kelly T.F., Miller M.K. (2007). Invited Review Article: Atom Probe Tomography. Rev. Sci. Instrum..

[16] Miller M.K., Babu S.S., Burke M.G. (2002). Comparison of the Phase Compositions in Alloy 718 Measured by Atom Probe Tomography and Predicted by Thermodynamic Calculations. Mater. Sci. Eng. A-Struct. Mater. Prop. Microstruct. Process..

[17] Kulawik K. (2017). The Influence of the Precipitates on Strengthening of Inconel 718 Superalloy at High Temperature. Ph.D. Thesis.

[18] von Harrach H., Klenov D., Freitag B., Schlossmacher P., Collins P., Fraser H. (2010). Comparison of the Detection Limits of EDS and EELS in S/TEM. Microsc. Microanal..

[19] ImageJ: Image Processing and Analysis in Java. https://imagej.nih.gov/ij.

[20] Ritchie N.W.M. (2010). Using DTSA-II to Simulate and Interpret Energy Dispersive Spectra from Particles. Microsc. Microanal..

[21] Ritchie N.W.M. (2009). Spectrum Simulation in DTSA-II. Microsc. Microanal..

[22] Ritchie N. (2023). NIST DTSA-II. https://www.nist.gov/services-resources/software/nist-dtsa-II.

[23] Gauvin R., Michaud P. (2009). MC X-ray, a New Monte Carlo Program for Quantitative X-ray Microanalysis of Real Materials. Microsc. Microanal..

[24] Drouin D., Couture A.R., Joly D., Tastet X., Aimez V., Gauvin R. (2007). CASINO V2.42-A Fast and Easy-to-Use Modeling Tool for Scanning Electron Microscopy and Microanalysis Users. Scanning.

[25] Geng W.T., Ping D.H., Gu Y.F., Cui C.Y., Harada H. (2007). Stability of Nanoscale Co-Precipitates in a Superalloy: A Combined First-Principles and Atom Probe Tomography Study. Phys. Rev. B.

[26] Miller M.K. (2001). Contributions of Atom Probe Tomography to the Understanding of Nickel-Based Superalloys. Micron.

[27] Ji Y.Z., Lou Y.C., Qu M., Rowatt J.D., Zhang F., Simpson T.W., Chen L.Q. (2016). Predicting Coherency Loss of Precipitates in IN718 Superalloy. Metall. Mater. Trans. A-Phys. Metall. Mater. Sci..

[28] Pennycook S.J. (1989). Z-Contrast Stem for Materials Science. Ultramicroscopy.

[29] Pennycook S.J., Berger S.D., Culbertson R.J. (1986). Elemental Mapping with Elastically Scattered Electrons. J. Microsc..

[30] Walther T. (2006). A New Experimental Procedure to Quantify Annular Dark Field Images in Scanning Transmission Electron Microscopy. J. Microsc. Oxf..

[31] Williams D.B., Carter C.B. (2009). Transmission Electron Microscopy: A Textbook for Materials Science.

[32] Lu P., Yuan R.L., Ihlefeld J.F., Spoerke E.D., Pan W., Zuo J.M. (2016). Fast Atomic-Scale Chemical Imaging of Crystalline Materials and Dynamic Phase Transformations. Nano Lett..

[33] Klenov D.O., Stemmer S. (2006). Contributions to the Contrast in Experimental High-Angle Annular Dark-Field Images. Ultramicroscopy.

[34] Stadelmann P. (2023). JEMS: A Program for the Simulation of Images and Diffraction Patterns in Electron Microscopy. https://www.jems-swiss.ch.

[35] Kirkland E.J. (2016). Computation in Electron Microscopy. Acta Crystallogr. A-Found. Adv..

[36] Barthel J. Dr. Probe-High-Resolution (S)TEM Image Simulation Software. http://www.er-c.org/barthel/drprobe/index.html.

[37] Barthel J. (2018). Dr. Probe: A Software for High-Resolution STEM Image Simulation. Ultramicroscopy.

[38] Lobato I. (2022). MULTEM Simulaton Software, Ivanlh20/Multem. http://github.com/Ivanlh20/MULTEM.

[39] Lobato I., Van Aert S., Verbeeck J. (2016). Progress and New Advances in Simulating Electron Microscopy Datasets Using MULTEM. Ultramicroscopy.

[40] Saito G., Yamaki F., Kunisada Y., Sakaguchi N., Akiyama T. (2017). Three-Dimensional Analysis of Eu Dopant Atoms in Ca-Alpha-SiAlON via through-Focus HAADF-STEM Imaging. Ultramicroscopy.

[41] Forbes B.D., D’Alfonso A.J., Williams R.E.A., Srinivasan R., Fraser H.L., McComb D.W., Freitag B., Klenov D.O., Allen L.J. (2012). Contribution of Thermally Scattered Electrons to Atomic Resolution Elemental Maps. Phys. Rev. B.

[42] Allen L.J., D’Alfonso A.J., Freitag B., Klenov D.O. (2012). Chemical Mapping at Atomic Resolution Using Energy-Dispersive x-Ray Spectroscopy. MRS Bull..

[43] Xu W., Dycus J.H., Sang X., LeBeau J.M. (2016). A Numerical Model for Multiple Detector Energy Dispersive X-ray Spectroscopy in the Transmission Electron Microscope. Ultramicroscopy.

[44] Lu P., Romero E., Lee S., MacManus-Driscoll J.L., Jia Q.X. (2014). Chemical Quantification of Atomic-Scale EDS Maps under Thin Specimen Conditions. Microsc. Microanal..

[45] Spence J.C.H., Taftø J. (1983). ALCHEMI: A New Technique for Locating Atoms in Small Crystals. J. Microsc..

[46] Jeong J.S., Odlyzko M.L., Xu P., Jalan B., Mkhoyan K.A. (2016). Probing Core-Electron Orbitals by Scanning Transmission Electron Microscopy and Measuring the Delocalization of Core-Level Excitations. Phys. Rev. B.

[47] Oxley M.P., Allen L.J. (1999). Impact Parameters for Ionization by High-Energy Electrons. Ultramicroscopy.

[48] Pennycook S.J. (1988). Delocalization Corrections for Electron Channeling Analysis. Ultramicroscopy.

[49] Allen L.J. (2017). Simulation in Elemental Mapping Using Aberration-Corrected Electron Microscopy. Ultramicroscopy.

[50] Chen Z., Taplin D.J., Weyland M., Allen L.J., Findlay S.D. (2017). Composition Measurement in Substitutionally Disordered Materials by Atomic Resolution Energy Dispersive X-ray Spectroscopy in Scanning Transmission Electron Microscopy. Ultramicroscopy.

[51] Kothleitner G., Neish M.J., Lugg N.R., Findlay S.D., Grogger W., Hofer F., Allen L.J. (2014). Quantitative Elemental Mapping at Atomic Resolution Using X-ray Spectroscopy. Phys. Rev. Lett..

[52] Lugg N.R., Kothleitner G., Shibata N., Ikuhara Y. (2015). On the Quantitativeness of EDS STEM. Ultramicroscopy.

[53] Chen Z., Weyland M., Sang X., Xu W., Dycus J.H., LeBeau J.M., D’Alfonso A.J., Allen L.J., Findlay S.D. (2016). Quantitative Atomic Resolution Elemental Mapping via Absolute-Scale Energy Dispersive X-ray Spectroscopy. Ultramicroscopy.

[54] Buffat P. (2004). Identification of Nano-Phases: Relative Merit of Local Analysis Techniques (HRTEM, Nanodiffraction, EDS/EELS).

[55] Dycus J.H., Xu W., Sang X., D’Alfonso A.J., Chen Z., Weyland M., Allen L.J., Findlay S.D., LeBeau J.M. (2016). Influence of Experimental Conditions on Atom Column Visibility in Energy Dispersive X-ray Spectroscopy. Ultramicroscopy.

